# Supplementary material for: MR-DoC2: Bidirectional Causal Modeling with Instrumental Variables and Data from Relatives
Source: Behav Genet. 2022 Nov 2;53(1):63–73. doi: 10.1007/s10519-022-10122-x (PMC9823046; doi:10.1007/s10519-022-10122-x)
Supplement: Supplementary file 1 — Supplementary material 1 (DOCX 18.6 kb) [file 10519_2022_10122_MOESM1_ESM.docx]

# Supplementary material

## Table S1. Exemplary *b_1_*, *b_3_,* *g_1_* and *g_2_* values and the power for estimating g1. High confounding (*ra*=*re*=.3), asymmetric AE (*a_1_* =.5, *e_1_* =.5, *a_2_* =.3, *e_2_*=.7). Highest power with combinations of positive b_1_, b_3_, with negative g_1_ or g_2_ values. Ncpg_1_, non-centrality parameter for rejecting g_1_ = 0;powg_1_, power for rejecting g_1_ = 0; Ph2.Ph1, $\boldsymbol{\beta}$^2^ of the regression of Ph2 on Ph1. Rmz1 and 2, phenotypic twin correlations.

| **b_1_** | **b_3_** | **g_1_** | **g_2_** | **rf** | **Ph2.Ph1** | **rmz1** | **rmz2** | **ncpg_1_** | **powg_1_** |
| --- | --- | --- | --- | --- | --- | --- | --- | --- | --- |
| 0.075 | 0.030 | -0.05 | 0.05 | 0.3 | 0.054 | 0.578 | 0.284 | 12.73 | 0.946 |
| 0.075 | 0.075 | -0.05 | 0.02 | 0.3 | 0.026 | 0.572 | 0.340 | 12.73 | 0.946 |
| 0.075 | 0.075 | -0.05 | 0.05 | 0.3 | 0.072 | 0.595 | 0.310 | 12.73 | 0.946 |
| 0.075 | 0.030 | -0.05 | 0.02 | 0.3 | 0.027 | 0.574 | 0.294 | 12.73 | 0.946 |
| 0.075 | 0.030 | -0.05 | -0.05 | 0.3 | 0.033 | 0.605 | 0.347 | 12.73 | 0.946 |
| 0.075 | 0.075 | -0.02 | 0.02 | 0.3 | 0.080 | 0.598 | 0.334 | 5.00 | 0.609 |
| 0.075 | 0.030 | -0.02 | 0.02 | 0.3 | 0.076 | 0.594 | 0.293 | 5.00 | 0.609 |
| 0.075 | 0.030 | 0.02 | -0.05 | 0.3 | 0.043 | 0.501 | 0.356 | 4.23 | 0.538 |
| 0.030 | 0.030 | 0.02 | 0.05 | 0.3 | 0.328 | 0.634 | 0.396 | 1.69 | 0.255 |
| 0.030 | 0.030 | 0.02 | 0.05 | 0.3 | 0.328 | 0.634 | 0.396 | 1.69 | 0.255 |
| 0.030 | 0.030 | 0.02 | 0.05 | 0.3 | 0.343 | 0.642 | 0.401 | 1.69 | 0.255 |
| 0.030 | 0.030 | 0.02 | -0.05 | 0.3 | 0.034 | 0.468 | 0.349 | 1.69 | 0.255 |
| 0.030 | 0.075 | 0.02 | 0.05 | 0.3 | 0.343 | 0.647 | 0.451 | 1.69 | 0.255 |
